# Immunotherapy for Lung Cancer: Has it Finally Arrived?

**DOI:** 10.3389/fonc.2014.00288

**Published:** 2014-10-22

**Authors:** Ahmed A. Mostafa, Don G. Morris

**Affiliations:** ^1^Department of Oncology, University of Calgary, Calgary, AB, Canada

**Keywords:** lung cancer, vaccines, immune checkpoint inhibitors, clinical trials

## Abstract

The possible link between infection/inflammation/immune activation and a cancer patient’s outcome from both a causative and outcome point of view has long been postulated. Substantial progress in the understanding of tumor-associated antigens/epitopes, immune cellular subpopulations, cytokine pathways/expression, the tumor microenvironment, and the balance between tumor-immune suppression and stimulation have been made over the past decade. This knowledge has heralded a new era of tumor immunotherapy utilizing vaccines, immune checkpoint inhibition, and oncolytic viruses. Despite significant progress in the molecular era now with targeted therapeutics such as EGFR tyrosine kinase inhibitors and ALK fusion protein inhibitors that have significantly improved the outcome of these specific lung cancer subpopulations, the overall 5 year survival for all non-small cell lung cancer (NSCLC) is still <20%. Unlike malignancies such as malignant melanoma, renal cell carcinoma, and neuroblastoma given their documented spontaneous remission rates lung cancer historically has been felt to be resistant to immune approaches likely related to an immunosuppressive tumor microenvironment and/or lack of immune recognition. Defining responding populations, understanding the mechanism(s) underlying durable immune responses, and the role of chemotherapy, radiation, oncolytic viruses, and other tumor disrupting agents in augmenting immune responses have led to improved optimization of immune therapeutic strategies. The purpose of this review is to focus on the recent advances in lung immunotherapy with an emphasis on recent clinical trials in the last 5 years in NSCLC.

## Introduction

Lung cancer is the number one cause of cancer mortality globally and has an estimated incidence of 1.3 million new cases every year (1). Approximately 80–85% of the newly diagnosed cases of lung cancer are non-small cell lung cancer (NSCLC) (adenocarcinoma, squamous carcinoma, and large cell carcinoma) and 15–20% small cell lung carcinoma. In the majority of cases, patients present with unresectable and/or non-curable disease (2). Locally advanced, good performance status NSCLC patients may be offered concurrent chemotherapy, radical radiotherapy, and/or surgery, with a resultant 8-month progression-free survival rate and <15% 5-year survival (3). Patients diagnosed with metastatic disease newer cytotoxic chemotherapies such as pemetrexed [17-month median overall survival (OS)] and treatment with molecularly targeted therapeutics for adenocarcinomas, such as next generation small molecules targeting the EGFR (24 months median OS) and ALK inhibitors (20 months median OS), the survival rate for advanced disease has improved only marginally (4–6). In the last decade, there has been a better understanding on how cancer interacts with the immune cells and the ways that the cancer have developed to evade the immune system, resulting in a new era of cancer immunotherapy protocols, which may aid in overcoming the limitations of conventional therapeutic strategies (7).

**Figure 1:**
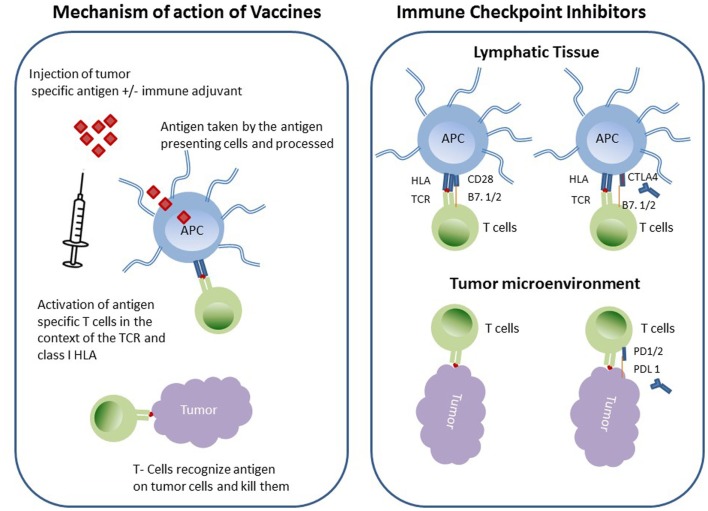
**Schematic diagram of immunotherapeutic strategies in NSCLC**.

Two such immunotherapeutic strategies in NSCLC are currently in clinical trials that involve increasing tumor immunogenicity by using cancer vaccines to augment tumor-immune recognition and overcoming tumor immunosuppression by using immune checkpoint inhibitors (Figure 1).

## Cancer Vaccines

Cancer vaccines are biologically active antigenic preparations that ideally educate the immune system about an existing cancer (8, 9). For a cancer vaccine to be effective, it should target an antigen specific to the cancer cell, i.e., tumor-associated antigens (TAA), which are frequently elevated in the circulation of cancer patients (10). Vaccines have historically been (glyco) peptides, recombinant proteins, or whole cancer cell preparations (that have been rendered replication incompetent); however, since antigenic peptides sub-optimally activate antigen presenting cells (APCs), vaccines are usually augmented by an immunoadjuvant or immunostimulant in the form of inactive pathogen or other non-specific immune stimulant. Cancer vaccines are taken up APCs, which later migrate to the nearest draining lymph node and consequently activate T- and B-lymphocytes. Specific T-cells will differentiate and expand to become tumor specific effector cells that will home to the tumor microenvironment which hosts the original antigens (11). It is of interest to speculate whether immune targeted therapeutics will be more effective if the tumor is initially disrupted by cytotoxic chemotherapy and/or radiation or some other cellular disrupting strategy, i.e., radiofrequency ablation/cryotherapy/oncolytic virus in order to augment antigen/epitope exposure to the immune system. There are numerous types of cancer vaccines that have been tested in clinical trials involving NSCLC patients as discussed below.

### Belagenpumatucel-L

Belagenpumatucel-L (Lucanix^®^) (NovaRx Corporation, San Diego, CA, USA) is an allogeneic tumor cell vaccine prepared from four irradiated human NSCLC cell lines SK-LU-1 (adenocarcinoma), NCI-H 460 (large cell carcinoma), NCI-H 520, and Rh 2 [squamous cell carcinoma, transfected with a transgene plasmid containing an antisense construct against the TGF-β2 gene (12)]. Elevated levels of TGF-β are frequently associated with immunosuppression in cancer through antagonizing the function of natural killer cells (NK) and dendritic cells (DC) (13). Moreover, the prognosis of NSCLC patients has been found to be inversely correlated with the level of TGF-β (14).

In the first of two phase II studies using the intradermal Lucanix vaccine involving stage II–IV NSCLC patients with low-tumor burden who had completed or refused conventional therapy tolerated the treatment well. Those patients with advanced disease who received higher doses (*N* = 41, >2.5 × 10^7^ cells/intradermal injection monthly) had a significant improved 2-year survival compared to the low-dose cohort [(*N* = 20, <2.5 × 10^7^ cells/injection) (47 versus 18%) (*p* = 0.0069)] (13). A second trial confined to pretreated stage IV NSCLC patients had an OS of 19 months (14). Interestingly, in this trial, the vaccine elicited both a cell mediated and a humoral immune response in the form of high level of cytotoxic cytokines and increased IgG and IgM titers.

The phase III survival, tumor free, overall, and progression-free (STOP) clinical trial, involving advanced NSCLC patients, pretreated with a first-line platinum-based chemotherapy, treated similarly (2.5 × 10^7^ cells/intradermal monthly injection) presented at the European Cancer Congress 2013 revealed a median OS of 20.3 and 17.8 months in Lucanix and placebo groups, respectively [hazard ratio (HR) 0.94; *p* = 0.594]. Although the OS was numerically longer STOP trial did not meet the primary endpoint. On the other hand, this analysis demonstrated improved OS in two subgroups, the non-adenocarcinoma and the stage IIIB/IV patients who started the vaccine therapy within 12 weeks of the finishing the initial chemotherapy.

### TG4010

The TG4010 vaccine is a suspension of recombinant modified vaccinia virus of Ankara (MVA strain) vector vaccine that expresses the TAA MUC1 and interleukin (IL)-2 (15). MUC1 is a transmembrane glycoprotein, which is normally expressed on normal duct epithelia, such as those lining the breast, prostate, lung, stomach, bladder epithelium, and sweat glands (16). Its normal function is related to mucin formation; however, in cancer its function is altered, due to excessive glycosylation, which contributes to its immunogenicity. High-MUC1 expression correlates with invasiveness and a poor prognosis for lung cancer (17). Furthermore, MUC1 overexpression activates phosphatidylinositol 3-kinase (PI3K) and the AKT pathways and resultant cell proliferation (18).

The initial randomized phase II study that included 65 patients with stage III/V NSCLC showed that TG4010 (10^8^ plaque forming units injected subcutaneously weekly for 6 weeks then every 3 weeks) in combination with chemotherapy (cisplatin/vinorelbine) (*N* = 44) versus TG4010 monotherapy until progression followed by the addition of chemotherapy (*N* = 21) was generally well tolerated. The combination group had a response rate of 30 versus 0% in the TG4010 group, however, a numerically inferior median and 1-year survival rate (19). Despite this a larger multicenter, open-label randomized phase IIB trial was conducted (20). This study enrolled 148 IIIB (pleural effusion)/IV patients with a 1:1 randomization to the combination therapy of TG4010 plus chemotherapy versus chemotherapy (cisplatin and gemcitabine) alone. The primary end point of the study was achieved with a resultant 6-month progressive free survival (PFS) of the combined group of 43% (95% CI 33.4–53.5) versus 35% (95%CI 25.9–45.3) in the control group. Notably, the objective response rate and median OS of responding patients was higher in the TG4010 group than in the chemotherapy alone group: 41.9 versus 28.4% and 23.3 versus 12.5 months, respectively. In this study, patients who presented with high levels for activation marker for NK cells (CD16^+^CD56^+^CD69^+^) at the baseline levels had the worst outcome. Thus, the presences of these markers may act as potential biomarker for the safety and efficiency of TG4010. Of note, FDA approved a phase III study on TG4010 in a subpopulation of patients with advanced NSCLC and normal levels of activated NK cells.

There is an ongoing Phase IIB/III randomized, double-blinded, placebo-controlled study comparing first-line therapy with or without TG4010 immunotherapy product in patients with stage IV NSCLC (TIME trial) currently accruing patients in Europe and the US.

### BLP25

BLP25 (Tecemotide^®^) (also known as L-BLP25 and Stimuvax) is a liposomal vaccine, which is formed from the immunoadjuvant monophosphoryl lipid A, and three lipid components (cholesterol, dimyristoyl phosphatidylglycerol, and dipalmitoyl phosphatidylcholine) (21) that harbors a 25 amino acid synthetic immunodominant core peptide of MUC1 TAA that has been shown to elicit a strong T-cell immune response both in transgenic murine lung cancer models and in patients (21–23). Recently, using a MUC1.Tg lung cancer mouse model, it was demonstrated that pre-administration of cyclophosphamide (CPA) with BLP25 increases the levels of the immune stimulating T-helper 1 (Th1) response [IL-2 and interferon gamma (IFN-γ)], as well as other inflammatory chemokines such as IP-10, MIG, KC, MCP-1, and MIP-1α (22), may enhance immunotherapy by boosting both cellular and humoral mediated antitumor immune responses for the vaccine by inhibiting regulatory T (Treg) cells (24–26).

A phase IIB clinical trial was conducted involving 171 stage IIIB and IV NSCLC patients with stable or better response to first-line chemotherapy or chemo-radiotherapy with a primary objective of OS and toxicity (21). The secondary endpoints investigated the health related quality of life (QQL) and immune response elicited by the vaccine. Patients were randomized to receive BLP25 plus best supportive care (BSC) versus BSC only. BLP25 or placebo was given subcutaneously weekly × 8 then 6 weekly until progression or significant toxicity. All patients in the BLP25 arm received a low dose of CPA prior vaccination. Although, the median survival time was 4.2 months longer in the treatment arm, this result was not statistically significant [17.2 versus 13 months, HR 0.74 (0.53–1.0)]. In addition, the 3-year OS was higher in BLP25 plus BSC group than the BSC group (31 versus 17%, *p* = 0.035). Interestingly, a 17.3-month improved survival as well as improved QQL was observed in those patients with stratified stage IIIB locoregional disease who received the BLP25 plus BSC [30.6 versus 13.3 months, HR 0.54 (0.3–0.99)]. The 3-year OS in this subgroup was also numerically higher in BLP25 plus BSC group than the BSC group (49 versus 27%, *p* = 0.07). Whether or not patients with a lower tumor burden, no metastasis and perhaps patients that receive multimodality treatment may benefit preferentially from this vaccine is unclear. Evidence of T-cell mediated immunity was only detected in only approximately 20% of the patients in the BLP25 arm, thought in part to be due technical problems related to decrease lymphocyte viability during collection and transportation.

On the strength of the above findings, two phase III trials were conducted. The stimulating targeted antigenic responses to NSCLC (START) clinical trial an international, randomized, double-blinded trial evaluated BLP25 as a maintenance therapy in stage III NSCLC patients with stable disease or better response after chemotherapy (27). The study was initiated in 2007 with recruitment of 1513 patients from 264 trial centers in 33 countries worldwide. Unfortunately, as a result of fatal encephalitis reported in a patient with malignant melanoma that was treated with BLP25 on an exploratory trial, the Food and Drug Administration agency placed a hold on the BLP25 clinical trials for approximately 135 days. This hold was suggested to have a negative impact on trial objectives as it resulted in a total of 274 patients from the BLP25 and placebo groups to be excluded from the study. The median OS and the 1–3 year survival rate between the two groups (BLP25 and placebo) were not statistically significant. Interestingly, the median OS for BLP25 compared to placebo arms in the concurrent chemo-radiotherapy subgroup was statistically significant [30.8 versus 20.6 months (HR 0.78, 0.64–0.95)]; however, no differences were noted in patients who had received sequential chemo-radiotherapy. The second phase II trial the INSPIRE trial (BLP25/Stimuvax trial **I**n Asian **NS**CLC **P**atients stimulating **I**mmune **R**esponse) (NCT01015443) is a double-blinded randomized 2:1 (BLP25: placebo) trial and is still ongoing (28). The study will target 420 patients with unrespectable stage III NSCLC from 40 trial sites in Asia (China, Hong Kong, Singapore, South Korea, and Taiwan) excluding Japan.

### MAGE-A3

Human melanoma antigen (MAGE)-A, -B, and -C are a family of genes normally expressed during embryogenesis and are also expressed in the immunoprivileged human tissues sites (29). Although, these genes are expressed in testicular germ cells and placenta trophoblasts, the antigens are not presented to the immune cells because of the lack of class I human leukocyte antigen molecules (HLA) (30, 31). For that reason, expression of these antigens on tumor cells that express class I HLA to the immune cells are likely immunogenic. Tumor cells such as melanoma, sarcoma, bladder, liver, esophageal, and lung cancers overexpress these antigens and hence considered tumor-associated antigens (32). MAGE-A3, a subtype of this family of genes, is differentially expressed in early stage (35%) and advanced stage (55%) lung cancer and hence it is theoretically a good target for tumor immunotherapy (33).

The MAGE-A3 vaccine (GlaxoSmithKline) is composed of the recombinant full-length protein MAGE-A3, *Haemophilus influenza* protein D that acts as an immune adjuvant and an immunostimulant AS02B or AS15 (34). The advantage of using the full protein is the production of several immunodominant epitopes that can be presented in the context of HLA class I and II and consequently activate both CD4 and CD8 T-cells. The broad array of T-cell responses can be in the form of Th response, cytotoxic T-cells (CTL), Th17 cells, and memory T-cells that result in immune effector antitumor immune responses (38). Recent findings indicate a beneficial role for MAGE-A3 vaccine in triggering the immune system including a study, which reported 84 genes as a gene expression signature (GS) in melanoma and NSCLC (35). These genes are involved in IFN-γ pathways, adaptive immunity, and specific chemokines that are responsible for T-cell activation and homing. When the MAGE-A3 vaccine was used with these GS-positive NSCLC patients, the disease free interval was in favor of the MAGE-A3 group compared to placebo group. In addition, no effect of the MAGE-A3 vaccine on the OS was noticed when GS was not taking into account, indicating that GS may act as an immune biomarker.

In order to evaluate the clinical benefit of the MAGE-A3 vaccine as an adjuvant treatment in postoperative lung cancer, 182 patients with completely resected MAGE-A3 positive stage IB/II NSCLC were enrolled into randomized (2:1 ratio), double-blinded, placebo-controlled phase II trial (36). Although all patients who received MAGE-A3 developed anti-MAGE-A3 immunoglobulin G antibodies, suggesting that vaccine triggered the immune response, no statistically significant difference were observed between the two groups with regards DFI, DFS, and OS. After applying forest plot analysis for HR (95% CI) to stratification factors, tumor stage, histology, and resection technique, all estimated values favored MAGE-A3 over placebo. Limited sample size and lack of chemotherapy as an adjuvant therapy were the main limitation of this study, which was later modified in the following phase III trial.

MAGRIT (**M**AGE-A3 as **A**djuvant **N**on-Small Cell Lun**G** Cance**R** Immu**noTh**erapy) was the largest ever phase III lung cancer adjuvant trial that aimed in determining the efficiency of MAGE-A3 vaccine as an adjuvant therapy following tumor resection in MAGE-A3 positive stage IB, II, and IIIA NSCLC (37). The other objectives were to study the toxicity. The study started in 2007 and recruited 2270 patient from 400 trial centers in 33 countries. Patients were randomly selected in 2:1 ratio and included patients who undergone surgery with or without adjuvant chemotherapy. Unfortunately, GlaxoSmithKline announced in April 2014 that MAGRIT study was to be discontinued due to failure to meet its primary objective, with no significant difference noted in DFS between MAGE-A3 and placebo group. Subgroup analyses are currently underway to see if there was a subpopulation that may have had more benefit.

### Other

There are many other vaccination strategies currently in preclinical or early human clinical trial testing. One of these utilizes the antigen PRAME (preferentially expressed in melanoma) involved in retinoic acid receptor repression although expressed in low levels in many normal tissues and is overexpressed in both melanoma and NSCLC and therefore a vaccination target. A dose escalation study of recombinant PRAME protein in a liposomal formulation containing the immune adjuvant AS15 (GSK2302032) is currently recruiting patients with resected early stage NSCLC. Other vaccines directed at epidermal growth factor ligand in combination with cyclophosphamide (CIMAvax) and cell therapy and oncolytic viral strategies containing constructs expressing various antigens or immune stimulating cytokines (GM-CSF) are currently being investigated.

## Immune Checkpoint Regulators

Initiation of adaptive immunity is a complex multifaceted mechanism that takes place between APCs and T-cells. A homeostatic balance between stimulatory and inhibitory signals is required to prevent over/under stimulation of T-cells, which may result in autoimmunity or immunosuppression sequelae, respectively (38, 39). APCs take up foreign antigen, process it, and express the antigen on its surface in the context of class II HLA, which then engages the T-cell receptor on the surface of T-cells. A second signal through the costimulatory molecules facilitated by binding of CD28 on T-cell surface by CD86 (B7-2) on APCs. As a result of these specific interactions, T-cells are activated and secrete cytokines (third signal) such as IL-2 stimulating T-cell clonal proliferation. In order to prevent autoimmunity, T-cell proliferation is negatively regulated by cytotoxic T-lymphocyte antigen 4 (CTLA-4), which is expressed on the surface of activated T-cells. CTLA-4 is a member of immunoglobulin superfamily and binds to B7-2 with much higher affinity than CD28 and therefore when expressed the T-cell response is down regulated. Furthermore, CTLA-4 is expressed by the Tregs thereby enabling them to suppress the effector T-cells. CTLA-4 regulation takes place in the early activation phase of immune induction occurring in the regional lymph nodes at the level of the APC and unprimed T-cell interaction.

Another significant immune check point regulator molecule that has been extensively studied is the programed death-1 (PD-1) molecule (40). PD-1 is expressed on the surface of activated T-cells and its active ligand [PD-L (B7-H1)] is expressed on macrophages and can be also actively induced in endothelial, epithelial, and tumor cells. PD-1 can also binds to PDL-2, which is expressed mainly on APC and some tumor cells. Unlike CTLA-4 negative regulation PD-1/PDL-1 takes place in the peripheral tissue/tumor during the effector phase of T-cell activation. Both CTLA-4 and PD-1 have been targeted by inhibitory antibodies as an adjuvant therapy in cancer in attempt to enhance T-cell activation and tumor immunity (41).

### Ipilimumab

Ipilimumab also known as MDX-010 and MDX-101 (Yervoy, Bristol-Myers Squibb) is a human monoclonal antibody directed against CTLA-4 molecule. Ipilimumab blocks the interaction of CTLA-4 with its ligand B7-2, resulting in T-cell activation, proliferation, induction of cytotoxic cytokines, and tumor suppression (42). Phase I/II trials have identified the safety and tolerability of CTLA-4 inhibition in several cancers that include the significant risk of colitis and, hepatotoxicity, skin rash, and hypophysitis/hypopituitarism (43). Moreover, they significantly improved OS in patients with malignant melanoma in phase III trials (44).

Two concurrent randomized phase II trials used ipilimumab in combination with chemotherapy (carboplatin/paclitaxel) for extensive stage small cell lung cancer (*n* = 130) and advanced stage NSCLC (*n* = 204) (45, 46). The primary endpoint of these studies was immune-related progression-free survival (irPFS). Secondary endpoints included PFS, best overall response rate (BORR), immune-related BORR (irBORR), OS, and safety. Patients were randomized to three groups (1:1:1), placebo/chemotherapy alone for up to six cycles, concurrent ipilimumab plus chemotherapy (four doses of ipilimumab/chemotherapy followed by two doses of placebo/chemotherapy) or phased ipilimumab (two doses of placebo/chemotherapy followed by four cycles of ipilimumab/chemotherapy). Phased ipilimumab, but not concurrent ipilimumab group, significantly improved irPFS in both the SCLC and NSCLC studies (HR 0.64 *p* = 0.03; HR 0.72, *p* = 0.05, respectively) and PFS in the NSCLC study (HR 0.69, *p* = 0.02) compared to patients who received placebo/chemotherapy alone. This finding was felt to be explained by chemotherapy induced tumor antigen release by chemotherapy trigger T-cell activation thus augmenting the effects of the immune checkpoint blockade (47). Of note, the improved irPFS in the phased ipilimumab NSCLC study was mainly confined to patients who had squamous cell histology. This is consistent with an increase T-cell infiltration found in squamous NSCLC (48). Further, an interesting case report of a patient with metastatic systemic treatment refractory NSCLC who was treated with palliative concurrent radiotherapy and ipilimumab that was associated with both a local and distant tumor complete response. A post-treatment increase in tumor-infiltrating cytotoxic lymphocytes, tumor regression, and normalization of tumor markers was observed. One year after treatment the patient was without evidence of disease based on PET/CT imaging (52). Two phase III trials NCT01450761 (ED-SCLC, etoposide/platinum, *N* = 1125, first data November 2015) and NCT01285609 (advanced NSCLC, carboplatin/paclitaxel, *N* = 920, first data October 2015) are still recruiting participants comparing ipilimumab plus chemotherapy versus chemotherapy alone in patients recently diagnosed ED-SCLC and squamous NSCLC, respectively.

### Nivolumab and MK-3475

Nivolumab (Bristol-Myers Squibb) and MK-3475 (Merck) are fully human antibodies that inhibit PD-1 receptors expressed on activated T-cells (49). Both block the binding of PDL-1/2 with PD-1 on surface of activated T-cells, and consequently increases T-cell activation by removing the inhibitory signaling of PD-1 (50). As PDL-1 is only expressed on selected tumor cells, the adverse effect of the drug is expected to be less than ipilimumab. A Phase I trial (*N* = 129) for nivolumab at three different doses (1, 5, and 10 mg/kg every 2 week) in NSCLC treatment refractory patients reported an overall 2 years survival rate 24% with median OS of 9.9 months with minimal toxicity (51). Interestingly, the 3 mg/kg group did the best with a BORR of 24.3% and a duration of response of 74 weeks and a median OS of 14.9 months. A Phase III trial involving nivolumab compared to docetaxel in second line and beyond is ongoing (NCT01673867) and will recruit 582 patients with metastatic/recurrent non-squamous NSCLC with a primary objective of OS in PD-1 inhibitor versus chemotherapy groups. The secondary objectives will determine PFS and disease related symptom progression, and evaluation of clinical benefit of PD-1 blocker. A second Phase III trial has just started accrual in advanced stage NSCLC PD-1 positive patients in first-line setting randomized to 3 mg/kg nivolumab every 2 week versus investigator choice chemotherapy. It is anticipated that 330 patients will be accrued to the study with a reporting date in 2017.

Merck also announced the result of phase Ib trial with a 24% immune-related response (IRRC), median OS was under a year and with minimal toxicity (49). Interestingly, 6/9 patients who met the IRRC had high levels of PDL-1, suggesting that this could be a predictor of response and survival. There are also six ongoing Phase I and Phase II studies involving PDL-1 blocking antibodies (MPDL3280A) in NSCLC.

## Summary

Targeting the immune system as a viable strategy for the treatment of lung cancer was until very recently not felt to be viable. Lung cancer historically was never felt to be a cancer histology that would lend itself to immune manipulation; however, we are now in an era of increased understanding of the complexity of tumor-immune interactions, which has facilitated over the past 5 years an increased interest and application of immune therapeutic strategies. The use of lung cancer directed vaccines and immune checkpoint inhibitors are driving these activities, however, in the future, it remains to be seen if tumor microenvironment cellular populations such as Tregs, myeloid derived suppressor cells (MDSC), tumor-associated macrophages, or soluble tumor immunosuppressive mediators such as indoleamine 2,3-dioxygenase (IDO), arginase, IL-6, IL-10, and other cytokines/chemokines will also be able to be targeted. Further, oncolytic viruses armed with immune stimulating constructs or in combination with immune checkpoint inhibitors, adoptive cellular therapies remain relatively untested in the clinic and are attractive to consider.

## Conflict of Interest Statement

The authors declare that the research was conducted in the absence of any commercial or financial relationships that could be construed as a potential conflict of interest.
